# Investigation of risk parameters in road accidents in Aras free zone 

**Published:** 2019-08

**Authors:** Mohammadsadegh Nafezi Alamdari

**Affiliations:** ^*a*^Aras Free Zone, Jolfa Safe Community. Jolfa, Iran.

## Abstract

The problem of deaths and injury as a result of road accidents is now acknowledged to be a global phenomenon. As a result authorities in virtually all countries of the world are now concerned about the growth in the number of people killed and seriously injured on their roads including Aras free zone. The objective of the paper was to identify the risk factors that mainly contribute to accident and their impact on road accident. This paper applied logistic regression to accident-related data collected Aras free zone police service, traffic records in order to examine the contributing factors to accident severity. A total of 494 accident data from 2007-2011 was used. The accident severity (dependent variable) in this paper is a dichotomous variable with two categories, Fatal or Non-fatal. Among the variables obtained from police-accident reports, five independent variables namely; gender, time of accident, location of accident and reasons assigned for accident were found to be statistically significant.

**Keywords:**

Accident Severity, Logistic Regression, Fatal Accident, Non-Fatal Accident

